# Immune system regulation and role of the human leukocyte antigen in posttraumatic stress disorder

**DOI:** 10.1016/j.ynstr.2021.100366

**Published:** 2021-07-12

**Authors:** Seyma Katrinli, Alicia K. Smith

**Affiliations:** a Emory University, Department of Gynecology and Obstetrics, Atlanta, GA, USA; b Emory University School of Medicine, Department of Psychiatry and Behavioral Sciences, Atlanta, GA, USA

**Keywords:** PTSD, Inflammation, Immune system, HLA, Major histocompatibility complex (MHC), Genomics, Epigenetics, Transcriptomics

## Abstract

Posttraumatic stress disorder (PTSD) is a debilitating condition that adversely affect mental and physical health. Recent studies have increasingly explored the role of the immune system in risk for PTSD and its related symptoms. Dysregulation of the immune system may lead to central nervous system tissue damage and impair learning and memory processes. Individuals with PTSD often have comorbid inflammatory or auto-immune disorders. Evidence shows associations between PTSD and multiple genes that are involved in immune-related or inflammatory pathways. In this review, we will summarize the evidence of immune dysregulation in PTSD, outlining the contributions of distinct cell types, genes, and biological pathways. We use the Human Leukocyte Antigen (HLA) locus to illustrate the contribution of genetic variation to function in different tissues that contribute to PTSD etiology, severity, and comorbidities.

## 1 Introduction

Posttraumatic stress disorder (PTSD) develops in some individuals that have experienced extreme, life threatening stress or trauma. PTSD is characterized by re-experiencing, avoidance and hyperarousal symptoms that cause negative alterations in cognition, mood, and physiologic health (Santiago et al., 2013; Thomas et al., 2010). Although the vast majority of the population is exposed to at least one traumatic event during their lifespan (Kessler et al., 1995; Breslau et al., 1998; Benjet et al., 2016), the prevalence of PTSD is 6.8% in US population (Kessler et al., 2005) and 3.9% globally (Koenen et al., 2017). This evident gap in prevalence between trauma exposure and PTSD development indicates that some individuals are more vulnerable to trauma and are at higher risk of developing PTSD.

A central question is why some individuals are more likely to develop PTSD compared to others after similar traumatic experiences (Nemeroff et al., 2006; Yehuda, 2004; Brewin and Holmes, 2003; Rothbaum and Davis, 2003). Heritability studies suggest that genetics contribute partially to the differential risk for PTSD following trauma (Koenen et al., 2002; Stein et al., 2002; Duncan et al., 2018). The recent meta-analysis from Psychiatric Genetics Consortium PTSD Workgroup (PGC-PTSD), which included 60 different studies with >200 K subjects, reported heritability (*h*^*2*^_*snp*_) in the range of 5–20%, with higher *h*^*2*^_*snp*_ in women compared to men, and significant shared liability between PTSD and other psychiatric disorders (Nievergelt et al., 2019). As growing genetic evidence highlights the associations between PTSD and genes with immune-related functions, ongoing research focus on the role of immune processes, including inflammation, in PTSD (reviewed in (Michopoulos et al., 2017; Hori and Kim, 2019; Sumner et al., 2020)).

In this review, we will outline associations between PTSD and inflammation, describing the contribution of the blood-based systemic immune response to neuroinflammation after stress or trauma exposure. We will focus on the role of immune-related genetic factors in PTSD, emphasizing variation in the Human Leukocyte Antigen (HLA) region in humans, also called the Major Histocompatibility Complex (MHC) in animal models. HLA genes regulate immune and inflammatory processes and were recently found to be involved in neuronal and synaptic plasticity, learning, memory, and behavior (Boulanger, 2009; Huh et al., 2000; Elmer and McAllister, 2012; Sankar et al., 2012; Yirmiya and Goshen, 2011). Understanding the genetic underpinnings of immune system dysregulation in PTSD has important translational and clinical implications that may promote testing of preventive approaches and treatment strategies.

## 2 Impact of stress on the immune system in PTSD

**Fig. 1:**
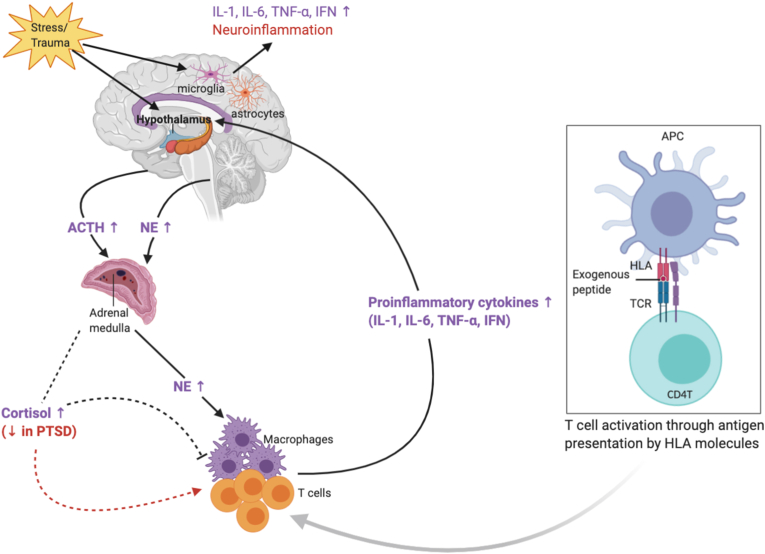
Possible mechanism by which immune activation contributes to inflammation in PTSD. Interactions between immune system, hypothalamic–pituitary–adrenal (HPA) axis and antigen presentation by HLA upon stress/trauma exposure is illustrated. ACTH, adreno-corticotropin; APC, antigen presenting cell; HLA, human leukocyte antigen; IFN, interferon; IL, interleukin; TCR, T cell receptor; TNF-α, tumor necrosis factor α. Adapted from Michopoulos et al., 2017 and Hori et al., 2019.

Dysregulation of the hypothalamus–pituitary–adrenal (HPA) axis in PTSD has been reviewed extensively (Fig. 1) (Hori and Kim, 2019; Schumacher et al., 2019; Michopoulos et al., 2016). Activation of the HPA axis results in hormonal and neurochemical alterations that signal back to the brain, such as elevation in blood levels of glucocorticoids, adrenaline, and norepinephrine. Repeated activation of the HPA axis by chronic stress and PTSD may cause dysregulated glucocorticoid signaling, increased inflammation in peripheral and central nervous systems, and ultimately, neuronal cell death and necrosis. The immune system is integral to these processes, as cellular corpses, neuritic debris, and neuronal cell remains cannot stay in the brain without interfering with its normal functioning (Yirmiya and Goshen, 2011). These neuro-hormonal processes also lead to peripheral immune activation as part of the body's natural fight-or-flight response. This acute inflammatory phase gradually resolves in healthy individuals. However, in some individuals with immune system dysregulation, the inflammatory response persists. Emerging studies have supported the potential for an immune-related or inflammatory etiology for PTSD and suggested that inflammation may be a preexisting vulnerability factor for the development of PTSD (Eraly et al., 2014; Cohen et al., 2011; Pervanidou et al., 2007).

### 2.1 Differences in immune cell distribution and function in PTSD

**Fig. 2:**
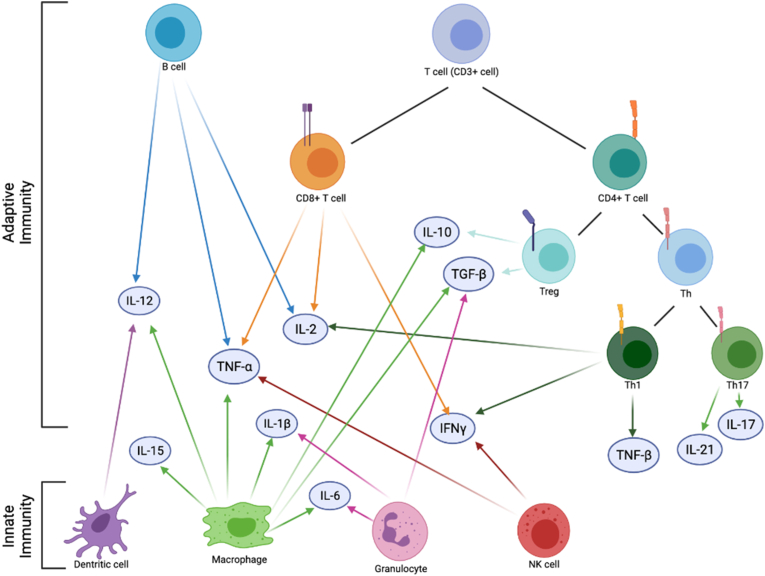
Delineated cells of the innate and adaptive immune system. For each cell type, the cytokines that have been implicated in PTSD are indicated. IFNγ, interferon gamma; IL, interleukin; NK, natural killer; TGF-β, tumor growth factor beta; Th, T helper cell; TNF-α, tumor necrosis factor alpha; TNF-β, tumor necrosis factor beta; Treg, regulatory T cell.

Immune system dysregulation in PTSD has also been supported by phenotypic or functional analyses of immune cell subpopulations (Fig. 2). Lymphocytes are central to the development of immune responses and comprise ~20–25% of the leukocyte population in peripheral blood. T-cells make up ~60–80% of lymphocytes and are defined by cluster of differentiation (CD) membrane co-receptors CD3 (i.e. CD3^+^ cells) (Chetty and Gatter, 1994). T-cells are further categorized by function, such as CD4^+^ T-helper (Th), immune inhibitory T-regulatory cells (Tregs), or cytotoxic CD8^+^ T-cells. CD4^+^ T-cells are involved in both the cell-mediated and humoral immune response through cytokine secretion (Fabbri et al., 2003). CD8^+^ T-cells are mainly responsible for destroying virally-infected cells and become memory cells upon encountering invading pathogens; they also contribute to cytokine production (Andersen et al., 2006). Cytokines are signaling molecules involved in the immune response as part of the innate immune system and are secreted by multiple cells, including peripheral immune cells (e.g., macrophages, lymphocytes), vascular endothelial cells, and central nervous system (CNS) members (e.g., microglia, astrocytes, and neurons) (Fig. 1) (Lacy and Stow, 2011; Galic et al., 2012).

Immunologic analysis of blood leukocytes in individuals with PTSD showed higher leukocyte, lymphocyte, total T-cell and CD4^+^ T-cell counts compared to trauma-exposed controls (Boscarino and Chang, 1999) (Fig. 2). Individuals with PTSD are reported to have reduced proportions of naïve CD8^+^ T-cells and Tregs, and increased proportions of CD3^+^ cells and memory (antigen-specific) T-cells (Sommershof et al., 2009). In a civilian cohort, PTSD cases had lower ratio of CD4^+^ T-cells to CD8^+^ T-cells and higher ratio of effector to naïve CD8^+^ T-cells (Aiello et al., 2016). This immune phenotype, referred as immune system aging, is generally observed in older individuals, as CD4^+^ T levels diminish and CD8^+^ T levels increase as individuals age (Pawelec, 2012). Hence, this evidence supports the association between PTSD and premature immune system aging (Lohr et al., 2015). PTSD is also associated with increased pro-inflammatory CD4^+^ T-cell subpopulations, Th1 and Th17 cells, and decreased Tregs that correlate with increased plasma interferon-gamma (IFN-γ) and Interleukin-17 (IL-17) levels (Zhou et al., 2014). A study of Croatian male combat veterans reported a lower proportion of CD4^+^ T-cells and Tregs in PTSD cases, indicating reduced capacity of immune suppression as a potential mechanism for enhanced immune reactivity (Jergovic et al., 2014). Since Tregs have anti-inflammatory properties and Th1 and Th17 cells have pro-inflammatory properties, skewing of immune cells towards Th17 or Th1 and away from Treg may be responsible for the chronic inflammatory state in PTSD (Jergovic et al., 2014). Together, this evidence suggests heightened lymphocyte activation in individuals with PTSD.

The above-described perturbations in the function and distribution of immune cells may explain PTSD-related alterations in blood inflammatory markers that have been reviewed extensively (Michopoulos et al., 2017; Hori and Kim, 2019; Sumner et al., 2020). Studies reported that individuals with PTSD are characterized by altered levels of C-reactive protein (CRP) (Eraly et al., 2014; Michopoulos et al., 2015; Lindqvist et al., 2017; O'Donovan et al., 2017; Miller et al., 2017) and peripheral cytokines, including, IFN-γ (Hoge et al., 2009; Lindqvist et al., 2014), interleukin-1β (IL-1β) (Passos et al., 2015), interleukin-6 (IL-6) (Lindqvist et al., 2017; Passos et al., 2015; Imai et al., 2018; de Oliveira et al., 2018) and tumor necrosis factor alpha (TNF-α) (Bruenig et al., 2017).

### 2.2 Immune genes associated with PTSD

CRP participates in the activation of the complement system. When stimulated by IL-6 or other proinflammatory cytokines, such as TNF-α and IL-1β, CRP activates the complement system, which triggers a series of events that promote inflammation (Diaz Padilla et al., 2003; Hovhannisyan et al., 2010). Multiple studies reported elevated serum CRP levels in individuals with PTSD (Michopoulos et al., 2015; Lindqvist et al., 2017; O'Donovan et al., 2017; Miller et al., 2001; Heath et al., 2013; Bersani et al., 2016), which led investigators to study gene polymorphisms in *CRP* as a candidate gene for PTSD. Michopoulos et al. showed that the *CRP* rs1130864 polymorphism is associated with elevated serum CRP levels and PTSD symptoms in a civilian African American population (Michopoulos et al., 2015). Moreover, Miller et al. reported that the *CR*P rs3091244 polymorphism moderates the association between lifetime trauma exposure and PTSD severity, whereas the *CRP* rs1205 and rs2794520 polymorphisms moderate the association between PTSD severity and serum CRP levels (Miller et al., 2018). Though multiple studies have investigated serum cytokine levels in PTSD patients (Hoge et al., 2009; Lindqvist et al., 2014; Miller et al., 2001; Bersani et al., 2016; Guo et al., 2012; Smith et al., 2011), only a few evaluated the genetic variations in cytokine genes. One such study, conducted on Vietnam War veterans, identified an association between a *TNF* SNP (rs1800629) and PTSD severity (Bruenig et al., 2017).

**Fig. 3:**
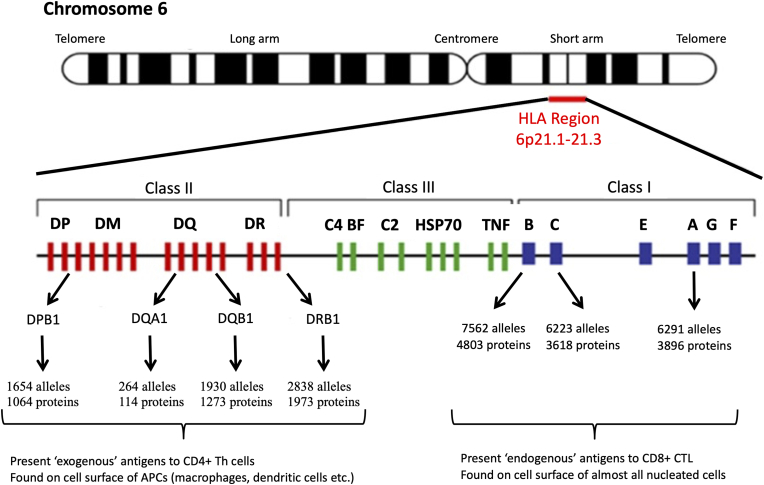
The genomic structure of the HLA region. The major genes encoded by the HLA region are illustrated. Class II genes (red) include: HLA-DPA1, -DPA2, -DPB1, -DPB2, -DMA, -DMB, -DOA, -DOB, -DRB1, -DRB2, -DRB3, -DRB4, -DRB5, -DRB6, -DRB7, -DRB8, and -DRB9. Class III genes (green) include: complement component C4A (C4A), complement component C4B (C4B), complement component C2 (C2), complement factor B (BF), Heat shock protein 70 (HSP70), and tumor necrosis factor (TNF). Class I genes (blue) include: HLA-B, –C, -E, -A, -G, and –F. For the most polymorphic genes, the number of named alleles and proteins is shown. APC, antigen presenting cell; CTL, cytotoxic T lymphocyte; Th, T helper. (For interpretation of the references to colour in this figure legend, the reader is referred to the Web version of this article.)

Transcriptomic studies have been used to examine differences in messenger RNA molecules expressed across all known genes. Transcriptomic studies of PTSD conducted in blood samples have reported alterations in expression of genes participating inflammatory pathways. *IL10* expression is upregulated in PTSD cases compared to controls (Mehta et al., 2018). Interestingly, a recent study that divided PTSD cases into those with high serum IL-6 levels and those with normal serum IL-6 levels, reported decreased *IL10* expression in PTSD cases with high serum IL-6 levels compared to controls with normal IL-6 levels (Hori et al., 2020). Studies have also reported upregulation of *CD7* and downregulation of Nuclear Factor 1 A (*NFIA*) in PTSD cases (Sarapas et al., 2011). Finally, expression of genes located in the HLA region (Fig. 3) have also shown to be associated with PTSD (Sarapas et al., 2011; Yehuda et al., 2009; Mehta et al., 2013). Consistent with these studies, gene-set enrichment analyses further implicate inflammatory and immune pathways in PTSD, including pathways involved in the immune response (Mehta et al., 2013; Neylan et al., 2011; Breen et al., 2015, 2018), cytokine-cytokine interactions (Mehta et al., 2018; Neylan et al., 2011), and the complement system (Bam et al., 2016a).

The PTSD-associated inflammatory gene expression changes reported are consistent with alterations in epigenetic profiles, as alterations in DNA methylation are capable of regulating gene expression and are observed in PTSD (Morrison et al., 2019). Functional annotation cluster analyses of uniquely unmethylated genes are enriched for inflammatory response, immune response, and innate immune response pathways in PTSD cases (Uddin et al., 2010). Individuals with PTSD have increased methylation at a CpG site located in Toll-like receptor 8 (*TLR8*) (Smith et al., 2011), and decreased methylation at CpG sites located in dedicator of cytokinesis 2 (*DOCK2*) (Mehta et al., 2017), nuclear factor of activated T-cells (*NFATC4*) (Hammamieh et al., 2017), *IL12B* (Bam et al., 2016b) and aryl hydrocarbon receptor repressor (*AHRR*) (Logue et al., 2020; Smith et al., 2020). Prospective studies investigating longitudinal methylation changes associated with PTSD implicated methylation changes in *HEXDC* and *HLA-DPB1* (Snijders et al., 2020), as well as enrichment in *IL17* signaling pathway (Rutten et al., 2018).

## 3 Large-scale genetic studies of PTSD implicate immune genes

Genome-Wide Association Studies (GWAS) are used to test the associations of common genetic variants across the whole genome with PTSD. These hypothesis-free large-scale genetic studies of PTSD identified variations in genes and pathways that participates in the regulation of immune system or that are associated with autoimmune and inflammatory disorders. A recent GWAS of PTSD conducted on Danish soldiers deployed to war and conflict zones identified a significant locus located downstream to the transcript stop position of the *IL-15*, highlighting a relation between deployment-related PTSD and inflammation (Wang et al., 2019).

Pathway analyses conducted using top GWAS hits also support the involvement of immune system in PTSD. Guffanti et al. identified 9 different modules of highly interacting genes, which showed enrichment for pathways involved in inflammation and autoimmune disorders, such as the antigen processing and presentation pathway and the Type I diabetes mellitus pathway (Guffanti et al., 2013). Ashley-Koch et al. conducted a pathway analysis of genes containing a SNP associated with PTSD at a nominal p-value < 0.001 across three ancestry-specific analyses (non-Hispanic white, non-Hispanic black, meta-analysis of non-Hispanic white and non-Hispanic black). The Gene Ontology (GO) analysis revealed that “Immunoglobulin I-set” was enriched in all three ancestry-specific analyses, supporting the role of the immune system in PTSD consistently among different ancestries (Ashley-Koch et al., 2015).

The recent PGC-PTSD GWAS identified a genome-wide significant hit (rs142174523) located on *HLA-B* in African American male participants across 13 cohorts (odds ratio = 0.76, p = 4.3 × 10^−8^) (Nievergelt et al., 2019). Expression quantitative trait locus (eQTL) analysis, which evaluated the functional role of this polymorphism on gene expression, suggested that rs142174523 regulates expression of 16 genes, including complement component C4A (*C4A*), complement component C4B (*C4B*), *HLA-B*, *HLA-C*, neurogenic locus notch homolog receptor 4 (*NOTCH4*), and psoriasis susceptibility 1 candidate 3 (*PSORS1C3*) (Nievergelt et al., 2019).

## 4 Human leukocyte antigen (HLA) and PTSD

The HLA region is one of the most extensively investigated and genetically diverse regions of the genome (Fig. 3). However, given the structural complexity and extensive polymorphism of the region, accurate interpretation of the HLA locus with standard GWAS methodologies is limited (Erlich, 2012). In addition, many of the most polymorphic regions of HLA genes are not well represented by high-throughput GWAS assays (de Bakker et al., 2006). The limitations of GWAS for fine-mapping of HLA variants led to the development of HLA imputation methods that can predict functional HLA alleles from GWAS data (de Bakker et al., 2006; Dilthey et al., 2013; Jia et al., 2013; Malkki et al., 2005; Zheng et al., 2014).

The specificity of an individual's immune response, transplant tissue compatibility, autoimmune disease risk (Matzaraki et al., 2017), and adverse drug reactions are all influenced, in part, by HLA molecules (Shiina et al., 2009). Growing evidence implicates the involvement of the HLA locus in psychiatric disorders, including schizophrenia (Purcell et al., 2009; Shi et al., 2009; Stefansson et al., 2009; Bergen et al., 2012; Andreassen et al., 2015), autism spectrum disorder (Johnson et al., 2009; Lee et al., 2006; Torres et al., 2002), major depressive disorder (Wray et al., 2018; Howard et al., 2018, 2019), and PTSD (Nievergelt et al., 2019; Katrinli et al., 2019). In this part, we describe the structure and function of the HLA locus, discuss some of the challenges for genetic analysis of the region, and describe some potential approaches for interpreting the role of the HLA region in PTSD etiology.

### 4.1 HLA structure and nomenclature

HLA complex is a 4 megabase (Mb) gene group found on the short arm of chromosome six (6p23.1) and represents ~0.1% of the human genome. The main role of HLA molecules is to present antigens to T cells (Trowsdale and Knight, 2013). In order to maintain a defense system for the diverse microbial environment, HLA genes must respond to a large number of highly variable antigens (Traherne, 2008). Hence, HLA genes are exceptionally polymorphic, with 28,320 allelic variants listed in the October 2020 release of the Immuno Polymorphism Database International ImMunoGeneTics project HLA Database (IPD-IMGT/HLA) (Robinson et al., 2020). Aside from structural complexity and extensive polymorphisms, the HLA region is also characterized by strong linkage disequilibrium (LD) among variants, usually at large distances (de Bakker et al., 2006). HLA alleles are combinations of different polymorphisms (i.e., haplotypes) that results in functionally distinct proteins. Hence, the nomenclature of the HLA region represents haplotypes, where each allele has its own unique number. The first two digits of the nomenclature (e.g., HLA-A*02, HLA-DQA1*01) describes the isoforms of HLA molecules, which are identified by antibody-based serotyping. Different HLA isoforms, represent variations in the exposed parts of the HLA protein, where antibodies bind. The four-digit coding captures nucleotide polymorphisms that change amino acid sequences, which defines functionally unique HLA alleles with distinct antigen presentation properties (e.g., HLA-A*02:01). This extensive variability of HLA alleles is the basis of individual immune responses.

The HLA region consists of 21 highly polymorphic genes characterized into three classes based on structure and function (Fig. 3). HLA class I genes (*HLA-A*, *HLA-B*, and *HLA-C),* located at the telomeric end of the region, are present on all nucleated cells and are responsible for presenting endogenous antigens to CD8^+^ T cells. HLA class II genes (*HLA-DRA, HLA-DRB1/3/4/5, HLA-DQA1, HLA-DQB1, HLA-DPA1,* and *HLA-DPB1*), found towards the centromeric region, are generally expressed in antigen presenting cells (e.g., macrophages and dendritic cells), and are responsible for presenting exogenous antigens to CD4+T cells. Class III genes that lie between these regions encode other non-HLA immune proteins, including components of the complement cascade, cytokines, heat shock proteins, transcription factors, other signaling molecules, transfer RNAs, and olfactory receptors (Robinson et al., 2013). In addition to their traditional immune functions, HLA Class I molecules are also involved in neurodevelopment, neuronal and synaptic plasticity, learning, memory, and behavior (Huh et al., 2000; Elmer and McAllister, 2012; Sankar et al., 2012; Yirmiya and Goshen, 2011). Particularly, HLA Class I genes show spatiotemporal expression during human hippocampal formation (Zhang et al., 2013) and regulate activity-dependent synaptic rearrangements in the developing and mature CNS (Huh et al., 2000; Corriveau et al., 1998). HLA Class I molecules also contribute to the normal developmental remodeling of glutamatergic synapses (Fourgeaud and Boulanger, 2010). Notably, HLA Class I molecules play an important role in behavior and stress reactivity. Genetically deficient MHC class I mice exhibited an enhanced behavioral response to acute stress (Sankar et al., 2012). Accordingly, expression of HLA genes was found to be altered in peripheral immune cells and in glial cells of those with psychiatric disorders (Jergovic et al., 2014; Bernstein et al., 2015; Goudriaan et al., 2014; Sinkus et al., 2013).

### 4.2 HLA alleles associate with PTSD

The diverse roles of HLA genes in the immune response as well as in the development, function, and integrity of the brain has made it an important molecule in biological psychiatry. The studies that have reported the association between PTSD and HLA genes are summarized in Table 1. A recent study reported associations between HLA alleles and PTSD (Katrinli et al., 2019). The investigators predicted 4-digit alleles of classical HLA genes (*-A, -B, -C, -DQA1, -DQB1, -DRB1, -DPB1*) in 403 PTSD cases and 369 trauma-exposed controls (Katrinli et al., 2019). In this study, eight HLA alleles were associated with PTSD after correction for multiple comparisons: HLA-B*58:01 (p = 0.035), HLA-C*07:01 (p = 0.035), HLA-DQA1*01:01 (p = 0.003), HLA-DQB1*05:01 (p = 0.009) and HLA-DPB1*17:01 (p = 0.017) were more common in PTSD cases, while HLA-A*02:01 (p = 0.026), HLA-DQA1*05:05 (p = 0.011) and HLA-DRB1*11:01 (p < 0.001) were more frequent in controls.

**Table 1:** HLA genes and alleles that associate with PTSD.

Study Type	Reference	Gene/Allele	Highlights
Genetics	Katrinli et al. (2019)	HLA-A*02:01	Less frequent in PTSD cases
		HLA-B*58:01	More frequent in PTSD cases
		HLA-C*07:01	More frequent in PTSD cases
		HLA-DQA1*01:01	More frequent in PTSD cases
		HLA-DQA1*05:05	Less frequent in PTSD cases
		HLA-DQB1*05:01	More frequent in PTSD cases
		HLA-DRB1*11:01	Less frequent in PTSD cases
		HLA-DPB1*17:01	More frequent in PTSD cases
Transcriptomics	Yehuda et al. (2009)	*HLA-DRB*	Decreased expression in PTSD
	Sarapas et al. (2011)	HLA Class II	Decreased expression in PTSD
	Mehta et al. (2013)	*HLA-A*	Increased expression in PTSD cases with childhood abuse
		*HLA-H*	Decreased expression in PTSD cases with childhood abuse
DNA Methylation	Snijders et al. (2020)	*HLA-DPB1*	Longitudinal methylation changes associated with PTSD
		*HLA-DRB1*	Longitudinal methylation changes associated with PTSD
	Katrinli et al. (2021)	*HLA-DPB1*	PTSD is associated with increased methylation in African Americans, and decreased methylation in Caucasians

Consistent with the genetic evidence, peripheral expression of HLA genes is altered in PTSD. A transcriptome-wide study conducted on 15 PTSD cases and 20 controls reported decreased *HLA-DRB* expression, along with reduced cortisol levels, in individuals with PTSD (Yehuda et al., 2009). Glucocorticoids induce expression of HLA class II genes in eosinophils (Schwiebert et al., 1995; Guida et al., 1994), but repress their expression in B cells (Celada et al., 1993). Hence, the dysregulation of the HPA axis in individuals with PTSD may disrupt the synergy between glucocorticoids and HLA class II genes, resulting in aberrant HLA class II gene expression. The association between PTSD and HLA class II gene expression was also replicated in a subsequent study in which Sarapas et al. reported lower HLA class II gene expression in current PTSD cases compared to controls and remitted PTSD (Sarapas et al., 2011). Moreover, when the effects of childhood maltreatment on PTSD-associated transcriptional profiles were evaluated, a total of 303 transcripts were differentially expressed between PTSD cases with childhood abuse vs. controls, and 244 transcripts were differentially expressed between PTSD cases without childhood abuse vs. controls after corrections for multiple testing. Notably, increased *HLA-A* expression was observed in PTSD cases with childhood abuse, while decreased *HLA-H* expression was identified in PTSD cases with childhood abuse (Mehta et al., 2013). Finally, Katrinli et al. evaluated the degree to which PTSD-associated HLA alleles influence gene expression (Katrinli et al., 2019), using weighted gene co-expression network analysis (WGCNA) (Langfelder and Horvath, 2008). The most intriguing gene expression module, which included pathways relevant for neural activity, was associated with HLA-C*07:01. This study supports that PTSD associates with HLA alleles with distinct gene expression patterns.

The PTSD-associated expression differences in HLA genes are consistent with reports from DNA methylation studies. A longitudinal meta-analysis of three male military cohorts reported that methylation levels across *HLA-DPB1* and *HLA-DRB1* regions are lower after deployment in individuals with PTSD (Snijders et al., 2020). However, a recent study reported that PTSD was associated with increased methylation across *HLA-DPB1* in a civilian cohort of predominantly African American women, whilst the association between PTSD and *HLA-DPB1* methylation was in opposite direction in a military cohort of predominantly Caucasian males (Katrinli et al., 2021). The difference in this direction of association may be explained by sequence-dependent DNA methylation patterns that are ancestry-specific, as the region includes methylation quantitative trait loci (meQTLs) that differ in minor allele frequencies (MAFs) by more than 40% between those of African versus European decent. Thus, given the extensive polymorphic variation in the HLA region, results from gene expression or DNA methylation studies are likely to be more consistent when interpreted along with information on allelic variation.

Since the HLA region is highly polymorphic and diverse, there are differences in the disease-associated alleles that exhibit ancestry-specific frequencies. Hence, sequencing of HLA alleles reflective of different ancestral backgrounds and studying the functional relevance of these alleles is crucial to be able translate findings from genomic studies into biological insight. The latter can be achieved by imputing functional HLA alleles in the larger datasets. The 4-digit coding groups HLA alleles according to the specific amino acid carried at each position. For some HLA alleles, the haplotype-specific amino-acid changes occur in the antigen-recognizing binding grooves, thereby influencing antigen recognition abilities. This is one possible mechanism to describe the role of HLA alleles in shaping the central and peripheral immune response that contributes to development or severity of PTSD (Fig. 1). Some HLA alleles with distinct amino-acid variations in their antigen-recognizing binding grooves have enhanced antigen presentation capacity, which result in abundant T cell activation. These activated T-cells induce production of pro-inflammatory cytokines, which may explain the pro-inflammatory milieu observed in individuals with PTSD. Similarly, specific HLA alleles have consistently been linked to autoimmune diseases, such as rheumatoid arthritis and multiple sclerosis, that co-occur with PTSD (Newton et al., 2004; Patsopoulos et al., 2013). Hence, genetic pleiotropy may provide insight into the shared etiology of these complex and comorbid disorders.

## 5 Conclusion and future directions

The heightened peripheral and CNS inflammation reported in those with PTSD may be, at least in part, determined by genetic variants involved in the immune response. Findings from genetic, transcriptomic, and epigenetic studies are inconclusive, but they are also promising, identifying similar genes across different types of study designs. Future studies in this area will benefit from studies with larger and more ancestrally diverse sample sizes. In addition, prospective studies are required to characterize the role of genetic variation on pathways and distinct cell types prior to the development of PTSD or its comorbid conditions. Longitudinal treatment studies with repeated assessments over time are also required to understand how the genetic and epigenetic regulation of the genes involved in immune system influence treatment response. Discovering the relationship between immune dysregulation and PTSD will likely contribute to research on potential anti-inflammatory treatments for individuals with PTSD.

## CRediT authorship contribution statement

**Seyma Katrinli:** Investigation, Writing – original draft, Visualization. **Alicia K. Smith:** Conceptualization, Writing – review & editing, Supervision.

## Declaration of competing interest

The authors do not have any competing financial interests related to the work described.
